# Hazardous alcohol use is associated with greater pain interference and prescription opioid misuse among persons living with HIV and chronic pain

**DOI:** 10.1186/s12889-021-10566-6

**Published:** 2021-03-22

**Authors:** Belle Ngo, Jane M. Liebschutz, Debbie M. Cheng, Jonathan A. Colasanti, Jessica S. Merlin, Wendy S. Armstrong, Leah S. Forman, Marlene C. Lira, Jeffrey H. Samet, Carlos del Rio, Judith I. Tsui

**Affiliations:** 1 University of Washington School of Medicine, Seattle, WA USA; 2 Division of General Internal Medicine, Department of Medicine, University of Pittsburgh School of Medicine, Pittsburgh, PA USA; 3 Department of Biostatistics, Boston University School of Public Health, Boston, MA USA; 4 Clinical Addiction Research and Education (CARE) Unit, Section of General Internal Medicine, Department of Medicine, Boston University School of Medicine, Boston, MA USA; 5 Division of Infectious Diseases, Department of Medicine, Emory University School of Medicine, Atlanta, GA USA; 6 Biostatistics and Epidemiology Data Analytics Center, Boston University School of Public Health, Boston, MA USA; 7 Department of Medicine, Boston University School of Medicine, Boston, MA USA; 8 Department of Community Health Sciences, Boston University School of Public Health, Boston, MA USA; 9 Department of Global Health, Rollins School of Public Health, Emory University, Atlanta, GA USA

**Keywords:** Pain, HIV, Opioids, Alcohol, Substance use

## Abstract

**Background:**

Alcohol use is common among persons living with HIV (PLWH), who often experience chronic pain, yet its impact on pain and opioid misuse is not fully characterized.

**Methods:**

We assessed associations between hazardous alcohol use and pain interference, defined as the self-reported impact of pain on daily living, pain severity, and risk for opioid misuse among PLWH who were on long-term opioid therapy (LTOT). A cohort was recruited as part of the “Targeting Effective Analgesia in Clinics for HIV” (TEACH) study, a randomized controlled trial to improve LTOT in HIV clinics. The Alcohol Use Disorders Test (AUDIT), Brief Pain Inventory (BPI) and the Current Opioid Misuse Measure (COMM) were administered at both baseline and 12-months. Linear mixed and generalized estimating equation models, incorporating data from both time points, evaluated associations between hazardous alcohol use (AUDIT ≥8) and: pain interference (0–10), pain severity (0–10), and opioid misuse risk (COMM ≥13), adjusting for age, gender, depressive symptoms, use of non-alcohol substances, time-point, and study-arm.

**Results:**

The sample was comprised of 166 participants, of which 31 (19%) reported hazardous alcohol use. The majority were male (65%), black (72%), and the mean age was 54 (range: 29–77). Hazardous alcohol use was significantly associated with higher pain interference (adjusted mean difference [AMD]: 1.02; 95% CI: 0.08, 1.96) and higher odds of opioid misuse risk (AOR: 3.73, 95% CI: 1.88–7.39), but not pain severity (AMD: 0.47, 95% CI: − 0.35, 1.29).

**Conclusions:**

Hazardous alcohol use was associated with greater functional impairment in daily living from their pain and higher odds for prescription opioid misuse in this study of PLWH on LTOT. Providers should be attentive to alcohol use among PLWH who are prescribed opioids given associations with pain and opioid misuse.

**Trial registration:**

ClinicalTrials.govNCT02564341 (Intervention, September 30, 2015) and NCT02525731 (Patient Cohort, August 17, 2015). Both prospectively registered.

## Background

Chronic pain is a major public health issue, affecting over 100 million Americans with annual costs over $600 billion [1–4]. The Center for Disease Control and Institute of Medicine of the National Academy of Sciences reports that chronic pain is a leading reason for adults seeking medical care and developing dependence to opioids [5]. The burden of pain is high among persons living with HIV (PLWH): a systematic review suggests that, on average, over half (54–83%) of all PLWH suffer chronic pain based on 3-month recall. As life expectancy for PLWH increases and the population of PLWH ages and develops chronic comorbidities, pain will play an even greater role in the future of HIV care such that the HIV Medicine Association of Infectious Diseases Society of America (IDSA) guidelines [6] now recommend screening all PLWH for chronic pain, as well as assessing the impact of pain on the activities of daily living (i.e. pain interference) through a multi-dimensional instrument such as the Brief Pain Inventory [6, 7]. The management of chronic pain among PLWH not infrequently includes prescriptions opioids [8], and prior research suggests that PLWH with chronic pain may be more likely to receive opioids compared to persons without HIV [9]. Given the emergence of the opioid use disorder epidemic in the U.S., it is important to understand factors that may lead to greater pain and opioid misuse in this vulnerable population.

Chronic pain and substance use frequently overlap in both HIV-infected [10, 11] and uninfected populations [12]. Substance use may be a maladaptive behavior to cope with pain [13, 14] and thus pain can serve as a trigger for use [15–17]. And yet substance use may also play a role in the generation or persistence of pain either indirectly through association with painful co-morbidities (e.g., secondary to trauma or neuropathy) or more directly as a consequence of withdrawal-related hyperalgesia. Acute alcohol consumption can have analgesic properties, [13, 18–20] but there is also evidence for hyperalgesia associated with alcohol withdrawal in both animal and human models [18, 20, 21]. Individuals with chronic pain may initially be motivated by these analgesic effects to “self-medicate” with alcohol, but subsequently develop increased reliance on alcohol for pain management leading to alcohol use disorders [13, 20, 22, 23]. Alcohol dependence and chronic pain share common neural circuits, which can become dysregulated and thus facilitate the transition of pain from an acute to chronic state [18, 20]. Thus alcohol may play an important role in the generation and maintenance of persistent pain [20, 24]. Furthermore, prior research conducted in non-HIV samples has shown that unhealthy alcohol use is associated with chronic pain severity [25] and interference [26]. Despite the fact that the prevalence of heavy drinking is twice that in persons with HIV than the general population [27, 28] and PLWH with chronic pain more often receive opioids [9], relatively little attention has been paid to relationships between alcohol, pain and opioid use in this population. The existing literature in the general population suggests that co-use of alcohol with opioids among persons with chronic pain is not uncommon and may contribute to opioid-related morbidity and mortality [29].

This study, through secondary analysis of data from a clinical trial, aims to characterize PLWH with chronic pain on long-term opioid therapy who reported hazardous alcohol use vs. those who did not report hazardous alcohol use, and to test the hypotheses that hazardous alcohol use is associated with pain interference, pain severity, and risk for misuse of prescribed opioids. The study leverages detailed data from a prospective cohort of PLWH with chronic pain who were on long-term opioid therapy as part of the “Targeting Effective Analgesia in Clinics for HIV” (TEACH) study, which include measures of hazardous alcohol use, pain and opioid misuse.

## Methods

### Study design

The study conducted secondary analysis using data from a prospective cohort of PLWH with chronic pain on long-term opioid therapy (LTOT) as part of the “Targeting Effective Analgesia in Clinics for HIV” (TEACH) study. The protocol fully describing study design, intervention, procedures, measure and planned analyses has been published, [30, 31], as well as the paper describing results [32]. Briefly, the study was a two-arm, unblinded cluster-randomized clinical trial to assess whether a collaborative care intervention improves guideline-concordant care compared to usual care for PLWH on LTOT, which included a parallel prospective patient cohort at both clinics with assessments at baseline and 12-months. The secondary analyses that comprise this study utilize data from this patient cohort only.

### Study participants

A cohort of PLWH receiving LTOT were recruited as part of the TEACH study between July 2015 and December 2016 from two safety-net, hospital-based HIV clinics in Boston and Atlanta [30]. Inclusion criteria for the TEACH cohort were as follows: age ≥ 18 years; HIV-positive; English-speaking; receiving LTOT (defined as having ≥3 opioid prescriptions written at least 21 days apart during the prior 6 months) [33]; and having completed a visit at the medical center’s enrollment sites at least once within the prior 18 months. Potential participants were identified through queries of the electronic medical record followed by review the medical records to confirm eligibility. Research staff contacted potential participants in person at the HIV clinics or by telephone to provide a study description and assess interest in participation. An invitation for a formal screening was offered to participants who were both eligible and interested. Secondary inclusion criteria included: 1) provision of contact information of 2 individuals to assist with follow-up; and 2) possession of a home or mobile telephone. Secondary exclusion criteria included: 1) plans to leave the area within the upcoming 12 months; and 2) inability to consent to or understand interviews [30].

### Study procedures

Two 60–90 min assessments were administered to the patient cohort participants by a research assistant (RA). After completing the first assessment, at baseline, participants were compensated with $35. Upon completion of the second assessment, 12-months post-baseline, participants were compensated with $50. The TEACH study was approved by the institutional review boards at Boston University Medical Campus and Emory University [30].

### Study measures

The main exposure of interest was past year hazardous alcohol use defined by a score of 8 or greater on the Alcohol Use Disorders Test (AUDIT) [34, 35]. The primary outcome of interest was past week pain interference as measured by the Brief Pain Inventory (BPI) [7, 36]. Pain interference was an average of seven questions asking participants to rate on a scale of 0–10 (0 = does not interfere; 10 = completely interferes) how much pain in the past week interfered with: general activity, mood, walking ability, normal work (both outside the home and housework), relations with other people and sleep. Secondary outcomes of interest were pain severity and risk for opioid misuse. Pain severity was measured using the average of four questions asking participants to rate from 0 to 10 (0 = no pain; 10 = pain as bad as you can imagine) their worst pain the past week, least pain in the past week, average pain in the past week, and current pain. Pain interference and severity measure separate constructs, and are each recommended as unique outcomes for clinical trials on chronic pain [37]. Risk for opioid misuse was measured using the Current Opioid Misuse Measure (COMM). The COMM™ is a seventeen-question patient self-report assessment of aberrant behavior related to opioids in the past 30 days. A threshold of ≥13 was utilized to indicate risk for opioid misuse based on a prior study demonstrating high sensitivity and specificity of predicting those patients with a prescription drug use disorder (PDD) [38]. Covariates used in descriptive analyses (of which some were included in regression models) were: demographics (age, gender, race/ethnicity); marital status; sexual orientation; housing status; employment status; prior history of incarceration; smoking status; HIV transmission risk category; self-reported hepatitis C status; number of years on chronic opioid therapy; morphine equivalent daily dose (MEDD) of current chronic opioid therapy; depressive symptoms; past 12-month use of substances other than alcohol; and report of any alcohol used in the prior 12 months. Depressive symptoms were measured using the Center for Epidemiologic Studies Depression Scale (CES-D) and a threshold of equal to or greater than 16 was used for presence of depressive symptoms [39, 40]. Use of substances other than alcohol was measured using the Addiction Severity Index which assessed past-30 day use [41]. Any alcohol was measured based on a single question asking “(In the past 12 months) how often do you have a drink containing alcohol?” with possible response categories being: (never, monthly or less, 2–4 times/month, 2–3 times/week, and 4 or more times/week). Any response greater than “never” was considered indicative of any alcohol use.

### Statistical analysis

Descriptive statistics characterized the sample overall and stratified by hazardous alcohol. Linear mixed effects models were used to evaluate the association between hazardous drinking and the outcomes pain interference (primary) and pain severity (secondary) across the study (i.e., repeated, cross-sectional analyses using data from both time points, baseline and 12-month follow-up). Generalized estimating equations (GEE) logistic regression models were used to assess the association between hazardous alcohol use and COMM score (dichotomized at ≥13) at both timepoints. The model used an exchangeable working correlation structure, and empirical standard errors are reported. These methods for correlated data account for incorporating repeated measures from the same individual from both baseline and 12 months. All models were adjusted for age, gender, depression, past 12-month use of substances other than alcohol, study time point, and RCT study participation/assignment (i.e., intervention group; control group; not in RCT). Potential confounders were selected a priori based on prior literature [42, 43]. Models were fully adjusted for all covariates simultaneously. Spearman correlation coefficients were assessed for independent variables and covariates and no pair of variables included in the regression models had a correlation > 0.40. Secondary analyses were conducted assessing any alcohol use, rather than hazardous use, as the exposure of interest. Two-tailed tests and a significance level of 0.05 were used for each analysis. All analyses were conducted using SAS 9.4 (Cary, NC).

## Results

Among the 166 participants included in the study sample, 65% were male, 72% black, and the mean age was 54 (range: 29 to 77) (Table 1). Twenty percent were employed part or full time, while 15% were homeless. At baseline, 15% (25/166) reported current hazardous alcohol use. Over the course of the study, 19% (31/166) reported hazardous alcohol, and 71% (118/166) reported any alcohol use. Overall mean pain interference was 5.6 (STD: 2.86, range: 0–10) and mean pain severity was 6.1 (STD: 2.44, range: 0–10). Thirty-four percent of people (57/166) reported COMM scores reflective of opioid misuse.

**Table 1:** Sample demographics and clinical factors overall and stratified by baseline hazardous alcohol use (*n* = 166)

	Hazardous Alcohol Use n (%)	No Hazardous Alcohol Use n (%)	Overall n (%)
**Total (N)**	25	141	166
**Mean age (± SD)**	54.5 (6.9)	53.8 (7.9)	53.9 (7.8)
**Gender**			
Male	15 (60.0%)	93 (66.0%)	108 (65.1%)
Female	10 (40.0%)	47 (33.3%)	57 (34.3%)
Other^a^	0 (0.0%)	1 (0.7%)	1 (0.6%)
**Race**			
White	3 (12.0%)	28 (19.9%)	31 (18.7%)
African American	22 (88.0%)	98 (69.5%)	120 (72.3%)
Other	0 (0.0%)	15 (10.6%)	15 (9.0%)
**Hispanic**			
Yes	2 (8.0%)	13 (9.2%)	15 (9.0%)
No	23 (92.0%)	127 (90.1%)	150 (90.4%)
Don’t know / refused	0 (0.0%)	1 (0.7%)	1 (0.6%)
**Married/living with partner**	6 (24.0%)	43 (30.5%)	49 (29.5%)
**Homeless**	7 (28.0%)	18 (12.8%)	25 (15.1%)
**Employed (part or full time)**	1 (4.0%)	32 (22.7%)	33 (19.9%)
**Incarcerated (past 12 months)**	1 (4.0%)	13 (9.2%)	14 (8.4%)
**Smokes cigarettes**	23 (92.0%)	68 (48.2%)	91 (54.8%)
**HIV transmission route**			
MSM/IDU	1 (4.0%)	8 (5.7%)	9 (5.4%)
MSM only	5 (20.0%)	37 (26.2%)	42 (25.3%)
IDU only	4 (16.0%)	16 (11.3%)	20 (12.0%)
Presumed heterosexual + blood/blood products	15 (60.0%)	80 (56.7%)	95 (57.2%)
**HCV co-infection**			
Yes	10 (40.0%)	43 (30.5%)	53 (31.9%)
No	14 (56.0%)	97 (68.8%)	111 (66.9%)
Don’t know / refused	1 (4.0%)	1 (0.7%)	2 (1.2%)
**Mean Duration of prescription opioid use**	5.3 (5.8)	7.3 (7.1)	7.0 (7.0)
**Depression**	15 (60.0%)	53 (37.6%)	68 (41.0%)
**Substance use other than alcohol (past 12 months)**	18 (72.0%)	64 (45.4%)	82 (49.4%)
**Mean pain severity score**	6.76 (1.93)	6.24 (2.20)	6.32 (2.17)
**Mean pain interference score**	6.71 (2.68)	5.75 (2.52)	5.90 (2.56)
**COMM score**			
Aberrant Use (COMM score ≥ 13)	12 (48.0%)	26 (18.4%)	38 (22.9%)
Non-aberrant Use (COMM score < 13)	12 (48.0%)	115 (81.6%)	127 (76.5%)
Don’t know / refused	1 (4.0%)	0 (0.0%)	1 (0.6%)
**Drinking of Alcohol**			
Never	0 (0.0%)	58 (41.1%)	58 (34.9%)
Any^b^	25 (100%)	83 (58.8%)	108 (65.0%)
**Morphine Equivalent Daily Dose (MEDD)**			
Mean (±SD)	25.51 (53.27)	37.06 (53.60)	35.32 (53.55)
25th, Median, 75th	0.00, 12.67, 23.00	4.20, 16.00, 40.00	4.00, 14.83, 36.46

Footnotes:

^a^ Self-reported male sex at birth

^b^ Includes those who responded yes to one of the following response options: Monthly or less, 2–4 times a month, 2–3 times a week, or 4 or more times a week

In the primary analysis, hazardous alcohol use was associated with greater pain interference (adjusted mean difference: 1.02; 95% CI: 0.08, 1.96), compared to no hazardous drinking (Table 2). The adjusted mean pain interference for the hazardous drinking group was 6.8 out of 10 compared to 5.8 for the not-hazardous drinking group. In secondary analyses, pain severity was not significantly associated with hazardous drinking (adjusted mean difference: 0.47; − 0.35, 1.29). In other secondary analyses that examined self-report of any drinking (rather than hazardous alcohol use), neither pain interference nor pain severity was significantly associated with any past 12-month drinking (adjusted mean difference: 0.20; − 0.56, 0.97 and − 0.30; − 0.96, 0.37, respectively). With regard to the association between drinking and prescription opioid misuse as measured by the COMM (Table 2), hazardous drinking was significantly associated with increased odds of medication misuse (AOR: 3.73, 95% CI: 1.88–7.39), while any past 12-month drinking was not significantly associated (AOR: 0.99, 95% CI: 0.49–1.99).

**Table 2:** Associations between alcohol use and pain interference, pain severity and having a Current Opioid Misuse Measure (COMM) score ≥ 13: results from unadjusted and adjusted mixed effects and GEE regression models ^a^

	Primary Outcome: Pain Interference^**b**^		Secondary Outcome: Pain Severity^**b**^		Secondary Outcome: COMM ≥ 13	
	Mean difference (95% CI)	*p*-value	Mean difference (95% CI)	*p*-value	OR (95% CI)	*p*-value
**Hazardous alcohol use**						
Unadjusted	1.43 (0.50, 2.36)	0.0029	0.74 (−0.07, 1.54)	0.07	4.22 (2.11, 8.44)	< 0.0001
Adjusted ^c^	1.02 (0.08, 1.96)	0.03	0.47 (−0.35, 1.29)	0.26	3.73 (1.88, 7.39)	0.0002
**Any alcohol use**						
Unadjusted	0.23 (− 0.48, 0.95)	0.52	− 0.21 (− 0.82, 0.39)	0.49	1.14 (0.65, 1.99)	0.65
Adjusted ^c^	0.20 (−0.56, 0.97)	0.61	−0.30 (− 0.96, 0.37)	0.38	0.99 (0.49, 1.99)	0.98

Footnotes:

^a^ 319 total observations were included in the models. Each of the 166 participants in this study could contribute a maximum of 2 observations (i.e. a baseline and follow-up observation). Due to missed follow-up visits for 13 participants, the total number of observations in the analyses was 319 rather than 332

^b^ Scale range 0–10

^c^Adjusted for age, gender, depression, other substance use, study visit, and study arm

## Discussion

In this cohort of PLWH with chronic pain on LTOT, hazardous alcohol use was associated with higher pain interference scores. Furthermore, participants with hazardous alcohol use had a greater than 3-fold increased odds for prescription opioid misuse risk as measured by a validated questionnaire. Pain severity did not appear to be significantly associated with hazardous alcohol use in adjusted or unadjusted models, but results should be interpreted cautiously due to the small sample size of the study. Although our study cannot establish causality, a possible explanation for these results may be that there are additional risks of drinking hazardous alcohol amounts among PLWH who are prescribed opioids for chronic pain. However, future studies are needed to confirm this.

This study builds upon prior research suggesting that hazardous alcohol use negatively contributes to patients’ pain experiences. A prior study conducted by Brennan et al. in HIV uninfected older adults showed that problem drinking was associated with greater pain severity and interference [13]. A large cohort study of 1514 people prescribed opioids for chronic non-cancer pain conducted by Larance et al. in Australia found that regular risky drinkers had significantly greater pain severity and interference compared to non-risky-drinkers; occasional risky drinkers also have greater pain interference, but not pain severity, compared to non-risky drinkers [26]. Greater pain among persons with hazardous alcohol use may be explained in part by overlapping neurocircuitry between alcohol use disorders and pain which allow heavy alcohol use to amplify subjective pain effects [18, 20, 24]. These research findings extend the literature on the impact of alcohol use on pain and opioid misuse to PLWH, a population that is at high at risk for chronic pain and receipt of LTOT. We observed that hazardous drinking was association with a 1-point mean increase in pain interference compared to those who did not report hazardous drinking, which is considered clinically significant in guidelines for clinical trial endpoints [44] and by other researchers [45]. This magnitude of effect is similar to that observed in the prior study by Larance et al. [26] In addition, this study supports the findings of a recent study which examined relationships between substance use and pain among PLWH using ecologic momentary assessments [46]. In that study alcohol emerged as the only substance that had a reciprocal relationship with pain: heavier drinking predicted worse pain and worse pain predicted heavier drinking. Interestingly, individuals in our study who had consumed alcohol within the past 12 months but who were not hazardous drinkers did not have the same risk of increased pain interference. Other research has also suggested that the adverse effects of alcohol on pain may not extend to moderate use [47]. These results reinforce clinical value for established thresholds of hazardous alcohol use, and bring to light a new complication of hazardous alcohol use, namely greater interference of pain on daily living. As it is not uncommon that patients will report alcohol use to cope with or self-medicate their pain, [13, 27, 48] it is important to make patients aware that heavy use is associated with worsening pain on their daily functioning.

Based on COMM scores, PLWH who had hazardous alcohol use also had significantly higher odds of medication misuse. This is consistent with the prior non-HIV focused literature which has demonstrated associations between binge alcohol use and prescription opioid misuse [49], as well as alcohol use disorders and nonmedical use of prescription drugs [50]. This may be due to the acute effects of alcohol use on judgment and executive function, which may lead to lack of control of opioid use, or individuals might be more likely to have a co-occurring opioid use disorder. Alternatively, hazardous drinking could potentially lead to more severe pain interference, which could create a perceived need for more frequent use or higher doses of opioids and subsequently translate into opioid misuse. A recent on-line survey study found that pain was associated with opioid misuse, but only among those with more severe alcohol use [51]. These results also make sense in the context of the strong relationship between chronic pain and substance use disorders, and speak to the greater risks for developing problems with prescribed opioids among persons with other co-occurring substance use disorders [52–57]. As such, it appears that judicious prescribing and careful monitoring of prescription opioids among PLWH who are hazardous drinkers is warranted. Additionally, providers should screen patients with chronic pain for hazardous alcohol use, especially among those on LTOT. In terms of interventions, there is good evidence that behavioral counseling interventions for risky or harmful alcohol use is effective in reducing this behavior among adults in primary care settings [58], although no specific therapies to address alcohol use in individuals on LTOT are considered standard of care. Research has demonstrated major gaps in adherence to guidelines for opioid prescribing among HIV providers [8, 59], despite the fact that PLWH report an awareness of the addictive potential for LTOT and report satisfaction with provider monitoring activities [31].

This study has limitations. The sample size was modest, and furthermore only a minority of participants had hazardous alcohol use. This limited our study power, and thus may explain why the association between hazardous alcohol and pain severity was not statistically significant. However, it is worth noting that that the prior mentioned study by Larance also found a more consistent impact of risky drinking on pain interference compared to pain severity [26]. Yet, the magnitude of that effect was also smaller than for pain interference, and therefore results might not be considered of clinical significance even if found to be statistically significant in a larger sample. In addition, given the observational study design, we cannot presume causality; to date the literature suggests bi-directional relationships between alcohol use and pain among PLWH [43, 46], which is consistent with the non-HIV focused literature [20, 47]. We acknowledge that it is possible that greater pain interference could lead to more heavy drinking, as prior studies have demonstrated associations between pain and heavy alcohol use [17] and relapse risk [15, 60]. Opioid misuse was measured by responses to a validated screening tool (COMM), which may not reflect actual behaviors and cannot be presumed to diagnose opioid use disorders. The study was based on secondary analysis of data collected as part of a clinical trial among PLWH on LTOT, conducted at two sites (i.e. Atlanta and Boston). The results may not generalize to other patient populations or other settings. Finally, strengths to this study include the prospective study design and use of validated instruments such as BPI, AUDIT and the COMM questionnaires.

## Conclusions

In summary, this study found that among PLWH on LTOT for chronic pain, hazardous alcohol use was significantly associated with higher pain interference and greater odds of opioid medication misuse based on an opioid risk screening tool. These results highlight the under-recognized contributions of a modifiable risk factor, hazardous alcohol use, and its association with increased pain on daily living and opioid misuse among PLWH on LTOT.

## Data Availability

The datasets used and/or analysed during the current study are available from the corresponding author on reasonable request.

## Abbreviations

PLWH: Persons living with HIV
LTOT: Long-term opioid therapy
TEACH Study: “Targeting effective analgesia in clinics for HIV” study
